# NIR light‐propelled bullet‐shaped carbon hollow nanomotors with controllable shell thickness for the enhanced dye removal

**DOI:** 10.1002/EXP.20210162

**Published:** 2022-11-18

**Authors:** Jinyang Lv, Yi Xing, Xiaoyu Li, Xin Du

**Affiliations:** ^1^ Beijing Key Laboratory for Bioengineering and Sensing Technology, School of Chemistry and Biological Engineering University of Science and Technology Beijing Beijing China; ^2^ National Engineering Laboratory for Hydrometallurgical Cleaner Production Technology Key Laboratory of Green Process and Engineering Institute of Process Engineering Chinese Academic of Sciences Beijing China; ^3^ University of Chinese Academy of Sciences Beijing China

**Keywords:** bullet‐shape, carbon hollow structure, dye removal, nanomotors, NIR‐laser propulsion

## Abstract

Materials with asymmetric nanostructures have attracted tremendous research attention due to their unique structural characteristics, excellent physicochemical properties, and promising prospects. However, it is still difficult to design and fabricate bullet‐shaped nanostructure due to its structural complexity. Herein, for the first time, we successfully constructed NIR light‐propelled bullet‐shaped hollow carbon nanomotors (BHCNs) with an open mouth on the bottom of nano‐bullet for the enhanced dye removal, by employing bullet‐shaped silica nanoparticles (B‐SiO_2_ NPs) as a hard template. BHCNs were formed by the growth of polydopamine (PDA) layer on the heterogeneous surface of B‐SiO_2_ NPs, followed by the carbonization of PDA and subsequent selective etching of SiO_2_. The shell thickness of BHCNs was able to be facilely controlled from ≈ 14 to 30 nm by tuning the added amount of dopamine. The combination of streamlined bullet‐shaped nanostructure with good photothermal conversion efficiency of carbon materials facilitated the generation of asymmetric thermal gradient field around itself, thus driving the motion of BHCNs by self‐thermophoresis. Noteworthily, the diffusion coefficient (*De*) and velocity of BCHNs with shell thickness of 15 nm (BHCNs‐15) reached to 43.8 μm⋅cm^−2^ and 11.4 μm⋅s^−1^, respectively, under the illumination of 808 nm NIR laser with the power density of 1.5 W⋅cm^−2^. The NIR laser propulsion caused BCHNs‐15 to enhance the removal efficiency (53.4% vs. 25.4%) of methylene blue (MB) as a typical dye because the faster velocity could produce the higher micromixing role between carbon adsorbent and MB. Such a smart design of the streamlined nanomotors may provide a promising potential in environmental treatment, biomedical and biosensing applications.

## Graphical abstract

This work describes the construction of bullet‐shaped carbon hollow particles with controllable shell thicknesses by heterogeneous interface growth on hard template. As a near infrared light‐propelled nanomotor, it shows higher diffusion coefficient and faster velocity by self‐thermophoresis for the enhanced dye removal.

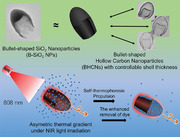

## 1 INTRODUCTION

The materials with asymmetric nanostructures have attracted tremendous attention in various application fields in recent years.^[^ ^1^, ^2^, ^3^, ^4^, ^5^ ^]^ A variety of asymmetric architectures have been created, such as bowl‐liked,^[^ ^6^, ^7^, ^8^, ^9^, ^10^ ^]^ bottle‐shaped,^[^ ^11^, ^12^, ^13^, ^14^, ^15^ ^]^ yolk‐shell,^[^ ^16^, ^17^, ^18^, ^19^, ^20^ ^]^ and others.^[^ ^3^, ^21^, ^22^, ^23^, ^24^, ^25^ ^]^ In addition, the components of materials with asymmetric nanostructures mainly include silica,^[^ ^26^, ^27^, ^28^ ^]^ carbon, and^[^ ^29^, ^30^, ^31^ ^]^ metals.^[^ ^32^ ^]^ For example, Kong's group fabricated asymmetric porous hollow carbon nanoparticles with a solid hemispherical shell and another porous hemispherical shell by kinetics‐controlled interfacial assembly method.^[^ ^33^ ^]^ Wang et al. reported hollow silica particles with flask bottle shape by a one‐pot soft template hydrothermal synthesis, which possessed a hydrophobic outer surface and another hydrophilic inner surface.^[^ ^11^ ^]^ Zhao's team synthesized the eccentric single‐pore mesoporous nanocages by developing an anisotropic encapsulation method.^[^ ^27^ ^]^ Compared with symmetric nanostructure, the asymmetric one should have unique properties of multiple functions and synergistic effects.^[^ ^34^ ^]^ Benefiting from these merits, these asymmetric nanomaterials display a promising potential in the applications of micro/nanomotors,^[^ ^35^, ^36^, ^37^, ^38^ ^]^ energy storage,^[^ ^39^, ^40^, ^41^, ^42^ ^]^ and catalysis.^[^ ^43^, ^44^, ^45^, ^46^ ^]^ Especially, Janus asymmetric micro/nanostructure is a significant foundation for the constructing of micro/nanomotors, which facilitates the generation of asymmetric field for efficient propulsion under the external stimulation.^[^ ^37^, ^47^, ^48^ ^]^

The design of streamlined structures of traffic tools in life, such as airplane, car, and high‐speed train, is in favor of their fast movement. Besides, the natural animals with streamlined body in water, including shark, sperms, and tadpoles, display tremendous velocity. Inspired by these objects in macrocosm, nanoparticles with streamlined structure may present enormous potential in reducing fluid or air resistance at the micro/nanoscale.^[^ ^49^, ^50^, ^51^ ^]^ For example, Yu et al. controllably synthesized a streamline shuttlecock‐shaped silica nanoparticle with a big opening on one side, which minimized the drag force during the fluid motion.^[^ ^24^ ^]^ Zhao's group fabricated a streamlined hollow mesoporous silica nanoparticle with a uniform tadpole shape, exhibiting fast diffusion behavior and obvious directional movement.^[^ ^49^ ^]^ Although some streamlined structured nanoparticles have been reported, it remains a great challenge to fabricate the streamlined nanoparticles due to their complex asymmetric structures up to now.

The driving velocity of micro/nanomotors can be affected not only by their own structure, but also by their own composition and the driving sources. Carbon‐based nanomaterials are considered as a promising choice to construct micro/nanomotors due to their outstanding physiochemical properties, such as excellent photothermal effect, low density, high stability, and biocompatibility.^[^ ^52^, ^53^, ^54^, ^55^, ^56^, ^57^ ^]^ These merits endow them with promising applications in gas and polluted water treatment, catalysis, biomedical, and so on.^[^ ^47^, ^58^, ^59^ ^]^ For the driving sources, light stimulation gradually becomes a promising choice to propel micro/nanomotor due to its high controllability, non‐pollution, and low‐cost.^[^ ^22^, ^35^, ^60^, ^61^, ^62^, ^63^ ^]^

Herein, we developed a NIR‐powered bullet‐shaped hollow carbon nanomotor with an open mouth on the bottom of nano‐bullet for enhanced MB removal. First, we prepared bullet‐shaped silica nanoparticles (B‐SiO_2_ NPs) by a dynamic hard template method via one pot synthesis strategy.^[^ ^11^ ^]^ Subsequently, as one of essential carbon precursors,^[^ ^64^, ^65^, ^66^ ^]^ polydopamine (PDA) was uniformly grown on the inner and outer surface of B‐SiO_2_ NPs, forming B‐SiO_2_@PDA NPs. Finally, bullet‐shaped hollow carbon nanomotors (BHCNs) were obtained by the carbonization of PDA, followed by the selective etching removal of B‐SiO_2_ NPs. Under 808 nm NIR laser illumination, the asymmetric thermal gradient field would be formed around streamlined BHCNs with asymmetric morphology, resulting in the self‐thermophoresis propulsion. The enhanced directional movement of BHCNs was applied to MB adsorption and removal.

## 2 MATERIAL AND METHODS

### 2.1 Chemicals

Polyvinylpyrrolidone (PVP, M.w.: 40,000 Da) and tris‐(hydroxymethyl)aminomethane were bought from Sigma Aldrich. *N*‐pentanol (99%), tetrabutylorthosilicate (TBOS, ≥98%), and hydrofluoric acid (HF) were provided by Shanghai Macklin Biochemical Co., Ltd. Trisodium citrate dihydrate was purchased from Sinopharm Chemical Reagent Co., Ltd. (3‐chloropropyl) trimethoxysilane (CPTMS, ≥98%) was purchased from Acros. Dopamine hydrochloride (99.9%) was purchased from Adamas‐beta reagent co., Ltd. Hexachloroplatinic (IV) acid hexahydrate (H_2_PtCl_6_·6H_2_O) and sodium borohydride (NaBH_4_) were purchased from Beihua Fine Chemicals. Aqueous ammonia (NH_3_·H_2_O, 25 ∼ 28%), absolute ethanol (99.5%), and methylene blue (MB) were obtained from Beijing Chemical Reagent Company. The water with a resistivity of higher than 18.2 MΩ·cm was produced by a Millipore Milli‐Q Plus 185 purification system.

### 2.2 Characterization

The detail parameters and description about sample characterization for transmission electron microscopy (TEM), scanning electron microscopy (SEM), X‐ray photoelectron spectroscopy (XPS), UV–visible absorption spectra, dynamic light scattering, and infrared thermal images were placed in Supporting Information.

### 2.3 Synthesis of B‐SiO_2_ NPs

The B‐SiO_2_ NPs were synthesized by hard template method according to the modified previous method.^[^ ^11^ ^]^ Typically, in a 100 ml of round‐bottom flask, 5.52 g of PVP was dissolved in 30 ml of *n*‐pentanol by sonication for 2 h. Then, 3 ml of absolute ethanol (99.5%), 1.2 ml of aqueous ammonia (NH_3_·H_2_O, 25 ∼ 28%), and 1170 μl of sodium citrate solution (0.03 mol⋅L^−1^) were mixed by sonication for a few seconds in a centrifuge tube. The above mixture was rapidly added to the PVP contained *n*‐pentanol solution under vigorous magnetic stirring (stirring bar with a size of 3 cm) at a speed of 900 rpm at room temperature. Next, the suspension was vigorously stirred at a speed of 900 rpm for 30 min. Subsequently, 600 μl of TBOS and 60 μl of CPTMS were added sequentially, and the mixture was stirred at a speed of 900 rpm for another 10 min. After reacting for 3.5 h under the static condition, the as‐synthesized B‐SiO_2_ NPs were obtained by centrifugation (11,000 rpm, 15 min), and then washed with ethanol and water for three times. The fabricated product was dried at 60°C for 12 h.

### 2.4 Synthesis of hydrophilic Pt NPs and their loading into B‐SiO_2_ NPs

50 ml of water and 100 μl of H_2_PtCl_6_·6H_2_O (0.4 mmol⋅L^−1^) were added to 100 ml of three neck round bottom flasks. After 26 ml of sodium citrate (2.8 mmol⋅L^−1^) was added to the above mixture, it was mixed by magnetic stirring with a speed of 400 rpm. 5 ml of freshly prepared aqueous NaBH_4_ (12 mmol⋅L^−1^) was then added dropwise under the stirring (stirring bar with a size of 4 cm, 400 rpm). In this process, the color of the mixed solution turned from pale‐yellow to dark‐brown in 5 min, and it continued to stir for 2 h. The as‐synthesized Pt NPs should be kept in a refrigerator at 4°C for subsequent use. 20 mg of B‐SiO_2_ NPs was mixed with 4 ml of hydrophilic Pt NPs by sonication for 30 min. After that, the mixture was separated via centrifugation (11,000 rpm, 15 min) to remove the unloaded Pt NPs.

### 2.5 Synthesis of B‐SiO_2_@PDA NPs

10 mg of B‐SiO_2_ NPs were added to 10 ml of Tris buffer (pH 8.5), and the mixture was sonicated for a few minutes. 5 mg of dopamine hydrochloride was then dispersed in the above suspension, and the formed mixture was stirred at 50°C for 24 h. B‐SiO_2_@PDA‐5 NPs were finally obtained by centrifugation (11,000 rpm, 15 min), and then washed with ethanol and water for three times. Under other identical conditions, B‐SiO_2_@PDA‐10 NPs and B‐SiO_2_@PDA‐15 NPs were prepared by regulating the amount of dopamine hydrochloride from 5 mg to 10 and 15 mg.

### 2.6 Synthesis of BHCNs

Typically, B‐SiO_2_@PDA NPs were calcined at 600°C for 2 h in N_2_ atmosphere. The calcined B‐SiO_2_@PDA NPs were then added to 2.4 ml of water, and the mixture was dispersed by sonication for a few minutes. Next, 800 μl of HF was mixed with the above suspension by sonication for 10 min to etch the silica component. The BHCNs were finally obtained by centrifugation (11,000 rpm, 15 min), and then washed with ethanol and water for three times.

### 2.7 Photothermal effect measurement

1 ml of water, B‐SiO_2_ NPs, B‐SiO_2_@PDA NPs, and BHCNs suspensions were added to 4 ml of centrifuge tubes, respectively. The particle concentration of three kinds of suspensions was ≈ 100 μg⋅ml^−1^. The centrifuge tubes were irradiated by an NIR laser with a wavelength of 808 nm (power: 1.0 W, light window: 3.14 cm^2^, laser from Hi‐Tech Optoelectronics Co., Ltd.) for 10 min. The distance between light window and centrifuge tube was fixed at ≈ 4 cm. In the heating process, the change of temperature was monitored by a digital thermometer with a 4‐channel data logger (RDXL4SD, Omega Engineering Inc.), and the head of the thermometer was immersed in the aqueous suspensions. The recorded time interval of temperature was 2 s during temperature‐rise period. The IR thermal images were pictured by using an infrared imaging device. The photothermal conversion stability of BHCNs‐15 (100 μg⋅ml^−1^) was tested via four cyclic tests. After BHCNs suspension was irradiated by 808 nm NIR laser (1.0 W⋅cm^−2^) for 10 min, the light source was turned off until the temperature of BHCNs‐15 suspension dropped back to room temperature. The four repeated cyclic processes were carried out in total.

### 2.8 Characterization of motion behavior of nanomotors

After B‐SiO_2_ NPs, B‐SiO_2_@PDA NPs or BHCNs aqueous suspension was added into a circular groove with a diameter of 5 mm and a depth of 0.5 mm on the quartz slide with a thickness of 2.5 mm, the groove was sealed with a high‐clean cover glass (8 mm of diameter) in order to minimize environmental disturbance. The beam collimator (2 cm of diameter) of 808 nm fiber‐coupled diode laser system was fixed at the same horizontal plane with quartz glass slide. Then, the switch of NIR laser was turned on to produce thermal gradient to trigger the movement of different NPs. After this irradiation process lasted for 20 s, the switch of NIR laser turned off. The motion of different NPs was measured under the irradiation of NIR laser with varied power density (0, 1.0, 1.5 W⋅cm^−2^). All the imaging experiments were recorded at a rate of 10 ∼ 12.5 frames⋅s^−1^ by employing a Nikon 80i darkfield upright microscope. This microscope was installed with a halogen tungsten lamp (100 W), an oil condenser (NA 1.20 ∼ 1.43), a plan fluor objective (40×/20×), and a charge‐coupled device camera (Olympus, DP73). The recorded images of motion behavior of the nanomotors were analyzed by using image processing program with ImageJ, and its MTrackJ plug‐in.

The effect of NIR laser irradiation on the UV–vis spectra of BHCNs aqueous suspension was investigated. 1 ml of BHCNs‐15 suspension (500 μg⋅ml^−1^) was added to a quartz cell. The area of lens flares of fiber‐coupled diode laser was consistent with that of BHCNs‐15 suspension in the quartz cell. After the BHCNs‐15 suspension was irradiated by NIR laser (1 W⋅cm^−2^) for different time (0, 5, 10, 20, 30 min), its UV–vis adsorption spectrum was measured. The placement direction of quartz cell was changed in order to tune the incidence direction of UV–vis spectrophotometer.

The effect of temperature on the UV–vis spectra was investigated. Typically, 1 ml of BHCNs‐15 suspension (500 μg⋅ml^−1^) was added to a quartz cell. After the quartz cell was placed in a water bath at 20°C for 5 min, its UV–vis absorption spectrum was measured. The quartz cell was then put back in the water bath. After water bath was heated to 30°C and kept at the temperature for 5 min, the quartz cell was taken out and measured by the UV–vis absorption spectrometer again. The same operation was carried out under different temperatures of 40°C, 50°C, and 60°C.

### 2.9 MB adsorption experiment

MB solutions with different concentrations (2, 5, 10, 20 μg⋅ml^−1^) were prepared to study the adsorption efficiency of BHCNs‐15. First, 150 μl of BHCNs‐15 (900 μg⋅ml^−1^) was added to 1800 μl of MB solution (20 μg⋅ml^−1^). Then, the adsorption of MB solution was measured under different conditions that the mixture was irradiated with or without NIR laser (1 W⋅cm^−2^) for 10, 20, and 30 min. At different time intervals, the supernatant of the mixture was obtained by centrifugation (12,000 rpm, 5 min), and was then measured by UV–vis adsorption spectrometer. The obtained absorbance data was used to determine the concentration of MB in the supernatant.

The following formulas were used to calculate the MB adsorption capacity in the adsorbent and its adsorption efficiency:^[^ ^65^, ^66^, ^67^ ^]^

(1) $$Q_{t}=\left(C_{0}-C_{t}\right)\times V/m$$

(2) $$R\left(\%\right)=\left(C_{0}-C_{t}\right)/C_{0}\times 100\%$$

where *C*_0_ and *C*_t_ are the MB concentrations (mg/L) at time 0 and *t* (min), respectively. *V* is the volume (L) of the MB solution, m is the weight (g) of the adsorbent, *Q*_t_ is the adsorption capacity (mg/g) at time *t* (min), and *R* is the MB removal percentage (%).

## 3 RESULTS AND DISCUSSION

### 3.1 Synthesis and characterization

**FIGURE 1:**
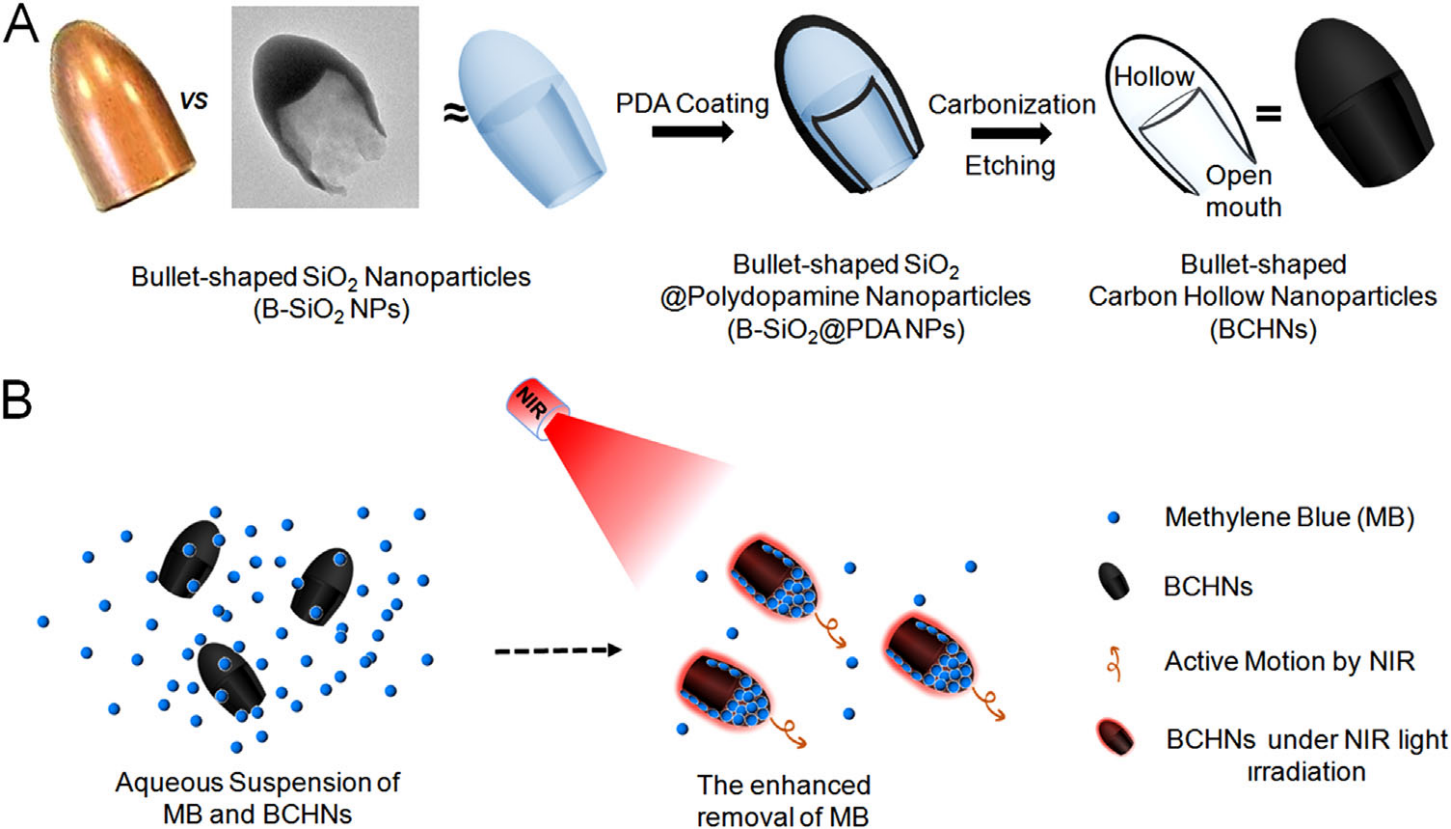
Schematic illustration for A the preparation process of BHCNs and B their NIR light propulsion for the enhanced MB removal

The fabrication process of BHCNs was exhibited in Figure 1A. First, B‐SiO_2_ NPs were fabricated by the modified dynamic hard template approach in one pot according to the literatures.^[^ ^11^, ^12^ ^]^ Second, dopamine hydrochloride was polymerized onto the surface of B‐SiO_2_ NPs in Tris‐HCl buffer (pH 8.5), forming PDA films on the inner and outer surfaces of B‐SiO_2_ NPs. The thickness of PDA films could be easily controlled by tuning the added amount of dopamine. Finally, B‐SiO_2_@PDA NPs were calcined in N_2_ atmosphere, followed by etched by HF solution to remove silica for the formation of BHCNs. In this procedure, PDA acted as the carbon precursor. After carbonization and removal of the internal silica, the obtained carbon materials still preserved the bullet‐shaped morphology and exhibited the hollow structure with an open mouth. Based on both the photothermal and dye adsorption properties of carbon materials, NIR light propulsion drove the faster motion of BHCNs by self‐thermophoresis, thus achieving the enhanced removal of organic dye such as MB, as shown in Figure 1B.

**FIGURE 2:**
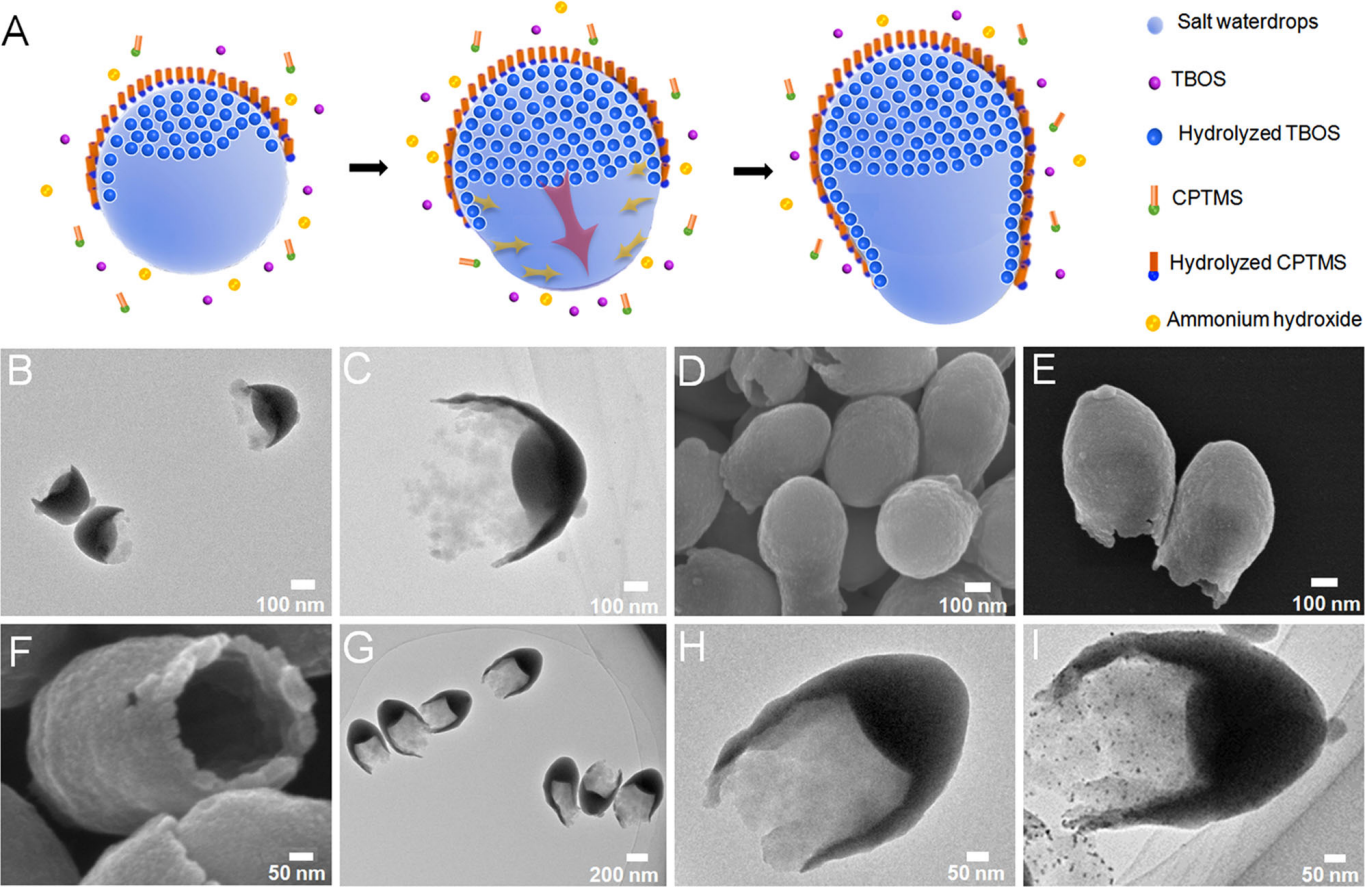
A,B TEM images of the morphology of B‐SiO_2_ NPs at reaction time of 2 h. C–E SEM images and F,G TEM images of the obtained B‐SiO_2_ NPs at reaction time of 3.5 h. H TEM image of hydrophilic Pt NPs loaded in the cavity of the B‐SiO_2_ NPs. I Schematic illustration of the possible growth mechanism of B‐SiO_2_ NPs

The morphology and structure of products were characterized by TEM and SEM. Figure 2A,B demonstrates the intermediate product at reaction time of 2 h during growth procedure (3.5 h) of B‐SiO_2_ NPs, which had a relatively small silica head and yet‐to‐be‐formed bullet‐shaped silica tail. For the finally obtained product after reaction for 3.5 h, as shown in SEM images (Figure 2C–E) and TEM images (Figure 2F,G), the synthesized product had a bullet‐like structure with an open mouth at the bottom of nano‐bullet, which was composed of a solid (nonporous) head and an open tail. The whole length size and width size of uniform B‐SiO_2_ NPs were ≈ 520 and ≈ 400 nm (Figure S1), respectively. The length of the hollow tail of B‐SiO_2_ NPs was ≈ 300 nm, and the length of the bullet head was ≈ 210 nm (Figure 2F,G). The open tail had a thin wall thickness of around 20 nm at the position of open mouth, and the end of hollow tail of B‐SiO_2_ NPs was rough and irregular (Figure 2E). The wall thickness of the hollow tail gradually decreased from the end of the bullet head to the end of the hollow tail (Figure 2G). In order to investigate the wettability of internal and external surface of B‐SiO_2_ NPs, hydrophilic Pt NPs were used as a probe. As shown in TEM image (Figure 2H), many hydrophilic Pt NPs were selectively loaded in the internal surface of hollow tail with an open mouth rather than its external surface, which proved the hydrophilicity of interior surface of B‐SiO_2_ NPs.^[^ ^11^ ^]^

We proposed a hypothesis about the growth mechanism of B‐SiO_2_ NPs in Figure 2I based on the above experimental phenomena and previous work.^[^ ^49^, ^50^, ^51^, ^68^ ^]^ At the beginning, the homogeneous water/*n*‐pentanol emulsion system was formed. When TBOS and CPTMS as silica precursors were added into the emulsion system, they hydrolyzed and condensed to form different silica species in alkaline conditions. Because the hydrolyzed TBOS species were hydrophilic, most of them stayed inside the water‐rich emulsion droplets to become silica nucleus, resulting in the formation of the initial head of B‐SiO_2_ NPs. While the hydrolyzed CPTMS species were amphiphilic, their hydrophilic component migrated toward the water phase and the hydrophobic section still remained in the oil phase. The hydrolyzed CPTMS species would form the main part of silica shell at the surface of emulsion droplets. With the extension of the reaction time, most of hydrolyzed TBOS species kept growing onto the existing silica nucleus, and this process gradually extruded the internal emulsion droplet to induce its deformation. In this dynamic deformation process of emulsion droplet, the existence of sodium citrate would regulate the interfacial tension between oil and water. The dynamic deformation of emulsion droplet should contribute to the tail formation of B‐SiO_2_ NPs. Because the volume of the added TBOS was 10 times that of CPTMS, the pure silica component from TBOS occupied the main one of B‐SiO_2_ NPs compared with amphiphilic organosilica from CPTMS.

**FIGURE 3:**
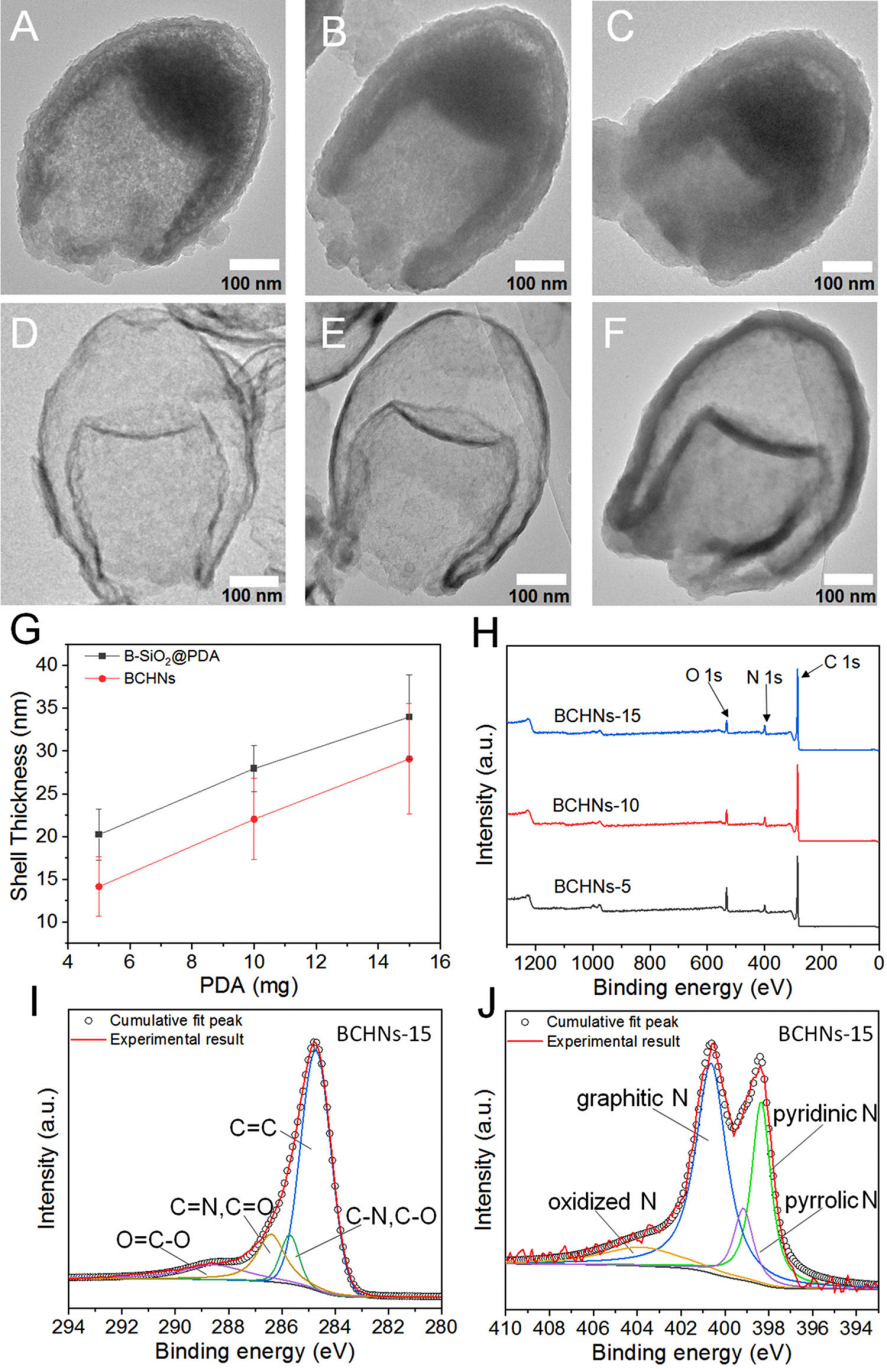
A–C TEM images of B‐SiO_2_@PDA‐X NPs prepared by different amounts of dopamine hydrochloride: A 5, B 10, and C 15 mg. D–F TEM images of BHCNs‐X. G Plot showing the dependence of shell thickness of B‐SiO_2_@PDA NPs and BHCNs on the added amount of dopamine hydrochloride. H XPS survey spectrum of BHCNs‐X, and high resolution XPS I C 1s and J N 1s spectra of BHCNs‐15

Next, B‐SiO_2_ NPs were employed as a hard template to grow PDA at their heterogeneous interface (Figure 1). PDA completely covered the internal and external surfaces of B‐SiO_2_ NPs in the form of a certain thickness, as shown in low (Figure S2A–C) and high (Figure 3A–C) magnified TEM images. With the increase of the added amount of dopamine from ≈ 5 to 10, then to 15 mg, the thickness of PDA layers increased from ≈ 20 to 28, then to ≈ 34 nm. The formed PDA layers were considered to be an excellent nitrogen–doped carbon source.^[^ ^64^ ^]^ After two steps of carbonization and etching, BHCNs were successfully fabricated, as shown in low (Figure S2D–F) and high (Figure 3D–F) magnified TEM images. Furthermore, it could be observed that BHCNs‐5 had a double‐shell structure, and their wall thickness decreased from ≈ 20 to only ≈ 14 nm due to the shrinkage caused by the pyrolysis of PDA (Figure 3D). Moreover, they preserved the intact bullet‐shaped morphology and structure, although their wall thickness was very thin. The bullet‐shaped structure with double‐shell suggested that the internal silica was removed and the carbon product was rigid. It is worth noting that the wall thickness of BHCNs could be easily tuned from ≈ 14 to 30 nm (Figure 3G) by controlling the thickness of PDA layer, as shown in TEM images (Figure 3D–F and Figure S2).

The XPS survey spectra of three BHCNs‐X showed three typical peaks of C 1s, N 1s, and O 1s in Figure 3H and Figure S3, indicating that the silica was completely removed. The C element was main component, while N element was the doped trace one. The high resolution C1s XPS spectrum showed four peaks at 284, 285.7, 287, and 289 eV, corresponding to C═C, C─N/C─O, C═N/C═O, and O═C─O, respectively (Figure 3I and Figure S3A,D,G). The peak of C═C was predominant, suggesting the existence of graphene structure in BHCNs‐X. The high resolution N1s XPS spectrum exhibited the existence of pyridinic N (398.5 eV), pyrrolic N (399.7 eV), graphitic N (401 eV), and oxidized N (402 eV) (Figure 3J and Figure S3B,E,H). The peaks of pyridinic N and graphitic N were main, which was consistent with those in the literatures.^[^ ^57^ ^]^

### 3.2 Photothermal effect

**FIGURE 4:**
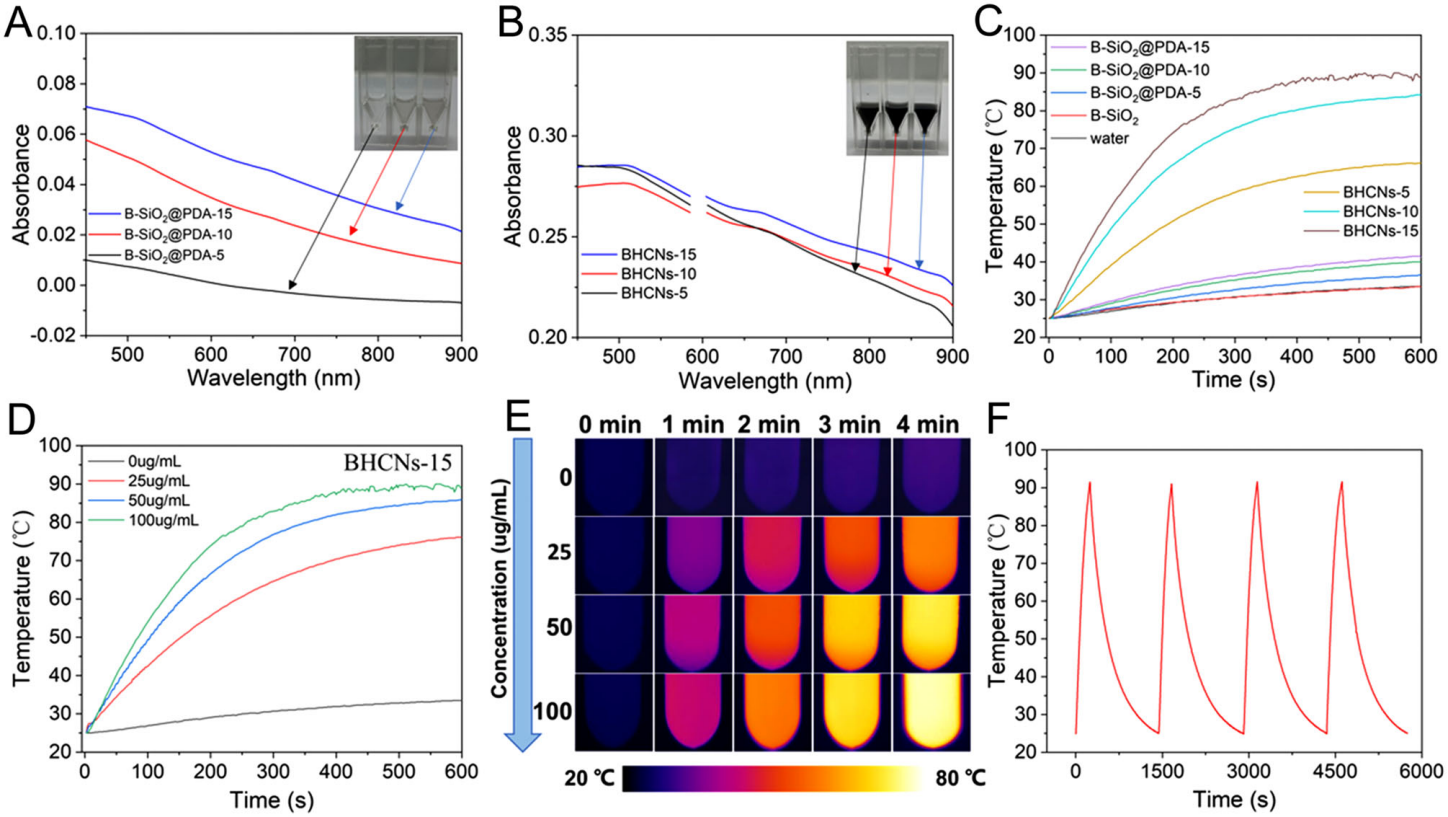
A UV–vis absorption curves of B‐SiO_2_@PDA‐X aqueous suspensions. B UV–vis absorption curves of BHCNs‐X aqueous suspensions. C Photothermal curves of water, B‐SiO_2_, B‐SiO_2_@PDA‐X, and BHCNs‐X suspensions under 808 nm NIR laser irradiation (1 W⋅cm^−2^) for 10 min. D Photothermal curves of BHCNs‐15 suspensions with different concentrations (0, 25, 50, and 100 μg⋅ml^−1^) under the irradiation of 808 nm NIR laser (1 W⋅cm^−2^) for 10 min. E Infrared thermal imaging photos of BHCNs suspensions with different particle concentrations (0, 25, 50, and 100 μg⋅ml^−1^) under the irradiation of 808 nm NIR laser (1 W⋅cm^−2^, 4 min). F The temperature change curve of BHCNs‐15 aqueous suspension (100 μg⋅ml^−1^) under 808 nm NIR laser irradiation (1 W⋅cm^−2^, 10 min) with light turned on/off for four cycles

The UV–vis absorption spectra of B‐SiO_2_@PDA‐X aqueous suspensions showed the enhanced light adsorption with the increase of thickness of PDA layer, which was agreement with the gradually darker color of corresponding suspensions (Figure 4A). As shown in Figure 4B, the color of BHCNs‐X suspensions had no obvious difference due to the black color of the carbon material. In addition, from the UV–vis absorption spectrum, it could be seen that the absorbance of BHCNs‐X suspensions was almost the same. The photothermal effect of B‐SiO_2_@PDA‐X and BHCNs‐X was exhibited in Figure 4C. The temperature of BHCNs‐5, BHCNs‐10, and BHCNs‐15 suspensions rapidly rose from 20.2°C to 66.2°C, 84.2°C, and 88.9°C, respectively, under the irradiation of NIR laser (wavelength: 808 nm) with the 1 W⋅cm^−2^ of power density for 10 min. In contrast, the temperature of B‐SiO_2_@PDA‐5, B‐SiO_2_@PDA‐10, and B‐SiO_2_@PDA‐15 suspensions only increased to 36.5°C, 40°C, and 41.5°C under the identical condition. Obviously, the photothermal effect of BHCNs‐X was much better than B‐SiO_2_@PDA‐X. It was mainly attributed to be that BHCNs‐X with black color had much higher light absorption property than B‐SiO_2_@PDA‐X with sepia color (Figure 4A,B), which should further result from the conjugation of benzene rings of the graphitized carbon of BHCNs‐X. Thus, BHCNs‐X could convert the NIR light energy into heat energy more effectively. The temperature rise of water and B‐SiO_2_ NPs suspension was only about 13°C, which proved the negligible impact on the photothermal effect. Figure 4D shows the temperature change curves of BHCNs‐15 with varied concentrations (0, 25, 50, and 100 μg⋅ml^−1^). Their temperature increased from 20.2°C to 33.5°C, 76.1°C, 85.9°C, and 88.9°C, respectively, at the corresponding concentrations, showing excellent photothermal effect. Infrared thermal images of BHCNs‐15 at four different concentrations under NIR laser illumination (808 nm, 1 W⋅cm^−2^, 4 min) displayed the obvious distinction as temperature rose (Figure 4E), which were in accordance with the heating curves of BHCNs‐15 during 4 min. Both heating curves and infrared thermal images revealed a relationship of particle concentration‐dependent temperature rising. Besides, after four NIR laser on/off cycles, no obvious change was observed for four heating curves, indicating the high stability of photothermal conversion (Figure 4F).

### 3.3 Self‐thermophoretic propulsion

**FIGURE 5:**
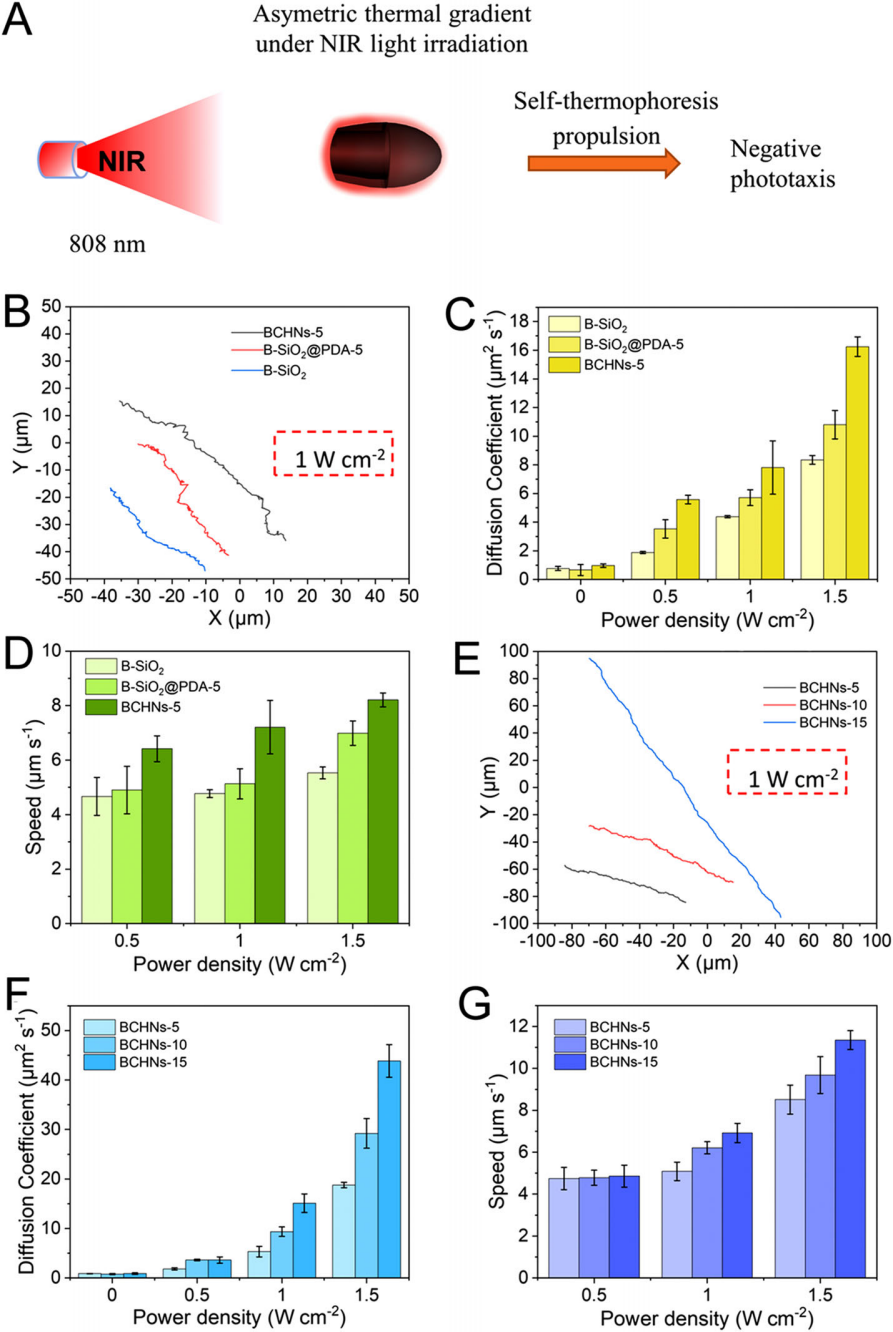
A Schematic illustration of the propulsion mechanism of bullet‐shaped nanomotors. B Trajectories of B‐SiO_2_ NPs, B‐SiO_2_@PDA‐5, and BHCNs‐5 nanomotors powered by 808 nm NIR laser (1 W⋅cm^−2^). C The *De* and D speed of B‐SiO_2_ NPs, B‐SiO_2_@PDA‐5, and BHCNs‐5 nanomotors under different power densities (0, 0.5, 1, 1.5 W⋅cm^−2^) of NIR laser. E Trajectories of BHCNs‐X nanomotors powered by 808 nm NIR laser (1 W⋅cm^−2^). F The *De* and G speed of BHCNs‐X nanomotors under different power densities (0, 0.5, 1, 1.5 W⋅cm^−2^) of NIR laser

Based on excellent photothermal conversion effect of BHCNs (Figure 4), we studied the NIR light‐propelled motion behavior of BHCNs. When NIR laser irradiated BHCNs from one side, the asymmetric thermal gradient would be generated owing to the asymmetric structure of bullet‐shape, thus driving the BHCNs to accelerate movement by self‐thermophoretic propulsion (Figure 5A). As shown in Figure S4A,E,I, the trajectories of Brownian movement of B‐SiO_2_ NPs, B‐SiO_2_@PDA‐5 NPs, and BHCNs‐5 displayed a typical random motion pattern. In contrast, under NIR laser illumination (808 nm) with different power densities (0.5, 1, 1.5 W⋅cm^−2^), the trajectories of B‐SiO_2_ NPs, B‐SiO_2_@PDA‐5 NPs, and BHCNs‐5 presented the directional movement (Figures S4A,E,I and 5B). Moreover, by altering power density of NIR laser from 0.5 to 1.5 W⋅cm^−2^, the trajectory lengths of three NPs not only obviously enhanced, but also turned directional. Both the corresponding effective diffusion coefficients (*De*) (Figure S4B,F,J) and speed (Figure S4C,G,K) increased with the increased power density. The mean‐square displacement (MSD) was also counted by the equation MSD = 4D·Δ*t* + v^2^·Δ*t* ^2^. The MSD curves of three particles displayed a parabolic increasing tendency with time interval (Δ*t*) under NIR irradiation (808 nm, 0.5, 1.0, 1.5 W⋅cm^−2^) (Figure S4D,H,L). It is worth noting that the trajectories length of BHCNs‐5 was obviously longer than those of B‐SiO_2_ NPs and B‐SiO_2_@PDA‐5 (Figure 5B and Video S1). The comparison of *De* among three particles was displayed in Figure 5C. The *De* of three particles notably increased under NIR laser irradiation with the increased power density (0.5, 1.0, 1.5 W⋅cm^−2^). Compare with B‐SiO_2_ NPs and B‐SiO_2_@PDA‐5 NPs, the *De* of BHCNs‐5 was dramatically increased as high as 16.3 μm^2^ s^−1^ under 1.5 W cm^−2^ of NIR laser irradiation. Correspondingly, the average velocity of BHCNs significantly increased to 8.2 μm⋅s^−1^ (Figure 5D). Compared with B‐SiO_2_ NPs and B‐SiO_2_@PDA‐5 NPs, BHCNs‐5 exhibited the enhanced directed movement under 808 nm NIR laser irradiation (Figure 5B–D), which should be ascribed to the best photothermal conversion ability (Figure 4C) and relatively low density of BHCNs‐5. In addition, it is interesting that B‐SiO_2_ NPs with main silica components displayed mild directed movement under NIR laser irradiation. It is well‐known that silica does not possess photothermal conversion property under NIR laser. The experimental results also showed that the raised temperature of pure water and B‐SiO_2_ NPs suspension was same under 808 nm NIR laser (Figure 4C). We proposed an assumption about this phenomenon: the thermal conductivities of SiO_2_ and water were 0.27e^−9^ W⋅(nm·K)^−1^ and 0.54 e^−9^ W⋅(nm·K)^−1^, and the thermal capacities of SiO_2_ and water were 966 J⋅(kg·K)^−1^ and 4186 J⋅(kg·K)^−1^, respectively.^[^ ^69^ ^]^ Due to the relatively large differences in thermal conductivity and thermal capacity between SiO_2_ and water, the temperature gradient might be generated under 808 nm NIR laser,^[^ ^69^ ^]^ driving B‐SiO_2_ NPs motion.

Furthermore, we also investigated the effect of morphology of nanoparticles on motion behavior under NIR light irradiation by comparing asymmetrically structured BHCNs‐5 with symmetrically structured hollow mesoporous carbon nanoparticles (HMCNs) (Figure S5A–D). The trajectory of HMCNs with uniform particle size (Figure S5B) exhibited the enhanced Brownian motion behavior under NIR laser, as shown in Figure S5C. However, the trajectory of BHCNs‐5 was linear and directional. Meanwhile, the *D_e_* of HMCNs was 1.9 μm^2^ s^−1^ under NIR laser (808 nm, 1.0 W⋅cm^−2^), which was lower than that (7.8 μm^2^⋅s^−1^) of BHCNs‐5 (Figure S5D). It was speculated that the asymmetric bullet‐liked morphology of BHCNs‐5 produced the enhanced asymmetric thermal gradient field around the nanoparticle compared with the symmetric spherical morphology of HMCNs, thus resulting in directional motion and faster movement speed.

Moreover, we investigated the effect of the thickness of carbon shell on the motion behavior. Figure 5E shows the trajectories of BHCNs‐X under 808 nm NIR laser irradiation with the power density of 1 W⋅cm^−2^. The trajectory length of BHCNs‐15 obviously was longer than those of BHCNs‐5 and BHCNs‐10. Moreover, the *De* and speed of three BHCNs‐X exhibited the gradually increased relationship dependent on powder density (Figure 5F,G and Videos S2 and S3). The *De* of BHCNs‐15 was calculated to be 43.8 μm^2^⋅s^−1^ under 1.5 W⋅cm^−2^ of 808 nm NIR laser, which was much higher than that (18.8 μm^2^⋅s^−1^) of BHCNs‐15 under the same condition (Figure 5F). When exposed to 1.5 W⋅cm^−2^ of 808 nm NIR laser for 20 s, the speed of BHCNs‐15 was computed to be 11.4 μm⋅s^−1^ (Figure 5G), which was higher than that (8.5 μm⋅s^−1^) of BHCNs‐5. With the increase of thickness of carbon shell of BHCNs, the enhanced motion was revealed under 808 nm NIR light irradiation, which should mainly result from the improved photothermal conversion effect (Figure 4C).

Compared with the results in the literatures, under 2.0 W⋅cm^−2^ NIR light irradiation, the *De* in the presence of 808 nm was 4.20 μm^2^⋅s^−1^,^[^ ^33^ ^]^ and the *De* under 2.5 W⋅cm^−2^ NIR irradiation only reached ≈ 3.49 μm^2^⋅s^−1^.^[^ ^70^ ^]^ In another report, the average speed of nanomotors reached 6.7 μm⋅s^−1^ under 1.96 W⋅cm^−2^ NIR laser.^[^ ^71^ ^]^ The *De* and speeds of these abovementioned nanomotors were obviously lower than 43.8 μm^2^⋅s^−1^ and 11.4 μm⋅s^−1^ of BHCNs‐15 under 1.5 W⋅cm^−2^ of 808 nm NIR laser. In short, these results demonstrated that 808 nm NIR laser could be used to achieve excellent self‐thermophoresis propulsion of BHCNs.

Next, we further investigated the phototaxis behavior of NIR light‐propelled BHCNs by controlling the incidence direction of UV–vis adsorption spectrophotometer and NIR laser illumination direction. The degree of decrease in absorbance upon two parallel directions (Figure S6A) was lower than those upon two vertical directions (Figure S6B). It suggested that the NIR laser triggered BHCNs to move toward the wall of cuvette and stick to it, that is, negative phototaxis. The effect of temperature was also studied as a control. BHCNs‐15 suspension was placed in the heating water, of which the temperature was changed from 20 to 60°C. The absorbance of BHCNs‐15 suspension had a bit smaller decrease than those obtained by NIR laser propulsion (Figure S6C), also suggesting that the motion of BHCNs was directional.

### 3.4 MB adsorption behavior of BHCNs

**FIGURE 6:**
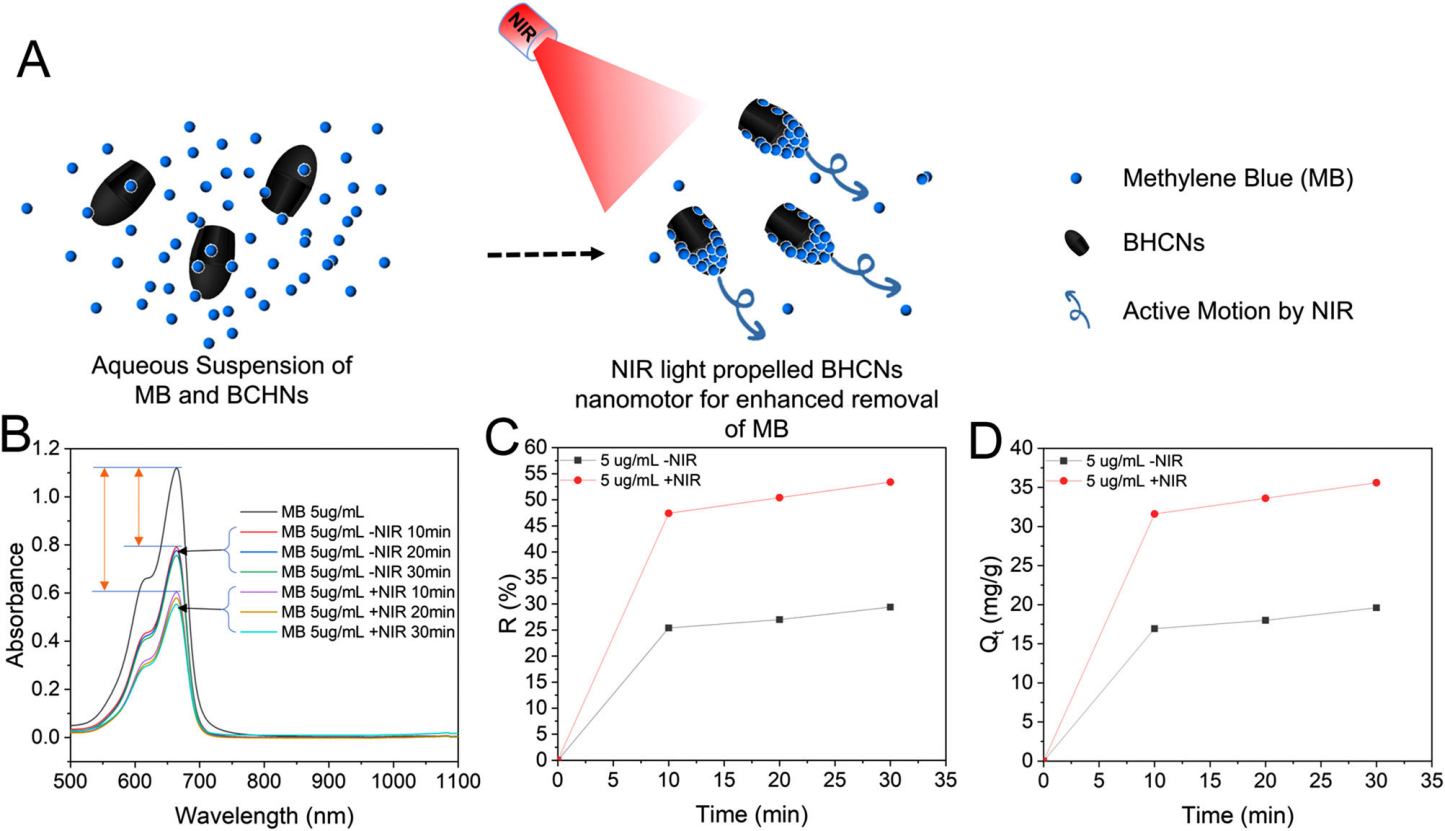
A Schematic illustration of BHCNs‐15 nanomotors powered by the irradiation of NIR laser for MB removal. B The UV–vis absorbance spectra of MB (5 μg⋅ml^−1^) under different conditions: static BHCNs‐15 micromotors (‐NIR) and dynamic BHCNs‐15 micromotors (+NIR). The orange double arrows indicated the degree of change in the absorbance of MB solution with or without NIR light irradiation. Time‐dependent changes of C R and D Q_t_ for MB (5 μg⋅ml^−1^) under different conditions: static BHCNs‐15 micromotors (‐NIR) and dynamic BHCNs‐15 micromotors (+NIR)

A set of experiments was implemented to investigate the removal efficiency of MB by employing BHCNs‐15 as an adsorbent with and without NIR laser irradiation (Figure 6A). As shown in Figure S7 and Figure 6B, for different concentrations (2, 5, 10, and 20 μg⋅ml^−1^) of MB solutions, all the absorbance values of MB in the supernatant under NIR laser irradiation were lower than those obtained without NIR laser irradiation. Moreover, when the concentration of MB solution was 5 μg⋅ml^−1^, BHCNs‐15 exhibited the biggest decrease under NIR laser illumination than those without NIR laser irradiation (Figure S7 and Figure 6B). To investigate the MB removal caused by photodegradation, control experiment was set up under the same condition. As shown in Figure S8, the absorbance values of MB solution under NIR laser irradiation with powder density of 1 W⋅cm^−2^ for 30 min only decreased a little (≈ 0.05), indicating that the photodegradation of MB under NIR laser irradiation with low powder density should be able to be ignored. The R and Q_t_ slightly increased with the extension of illumination time of NIR laser (Figure 6C,D). The R of MB was 53.4% under NIR laser irradiation for 30 min, much higher than that (25.4%) without NIR laser illumination (Figure 6C). The Q_t_ also dropped from 35.6 to 19.6 mg⋅g^−1^ with or without NIR laser. These results suggested that the motion effect of BHCNs was in favor of the enhanced MB removal (Figure 6D). It might be ascribed to the mechanism that the increases of speed of light‐driven BHCNs should affect the probability of contact between carbon adsorbent and MB dye. Under appropriate MB concentrations, it might result in a higher micromixing role between carbon adsorbent and MB for more efficient MB adsorption.^[^ ^72^ ^]^ These results suggested that the NIR laser propulsion was beneficial to enhance the removal of MB by using BHCNs as an adsorbent.

## 4 CONCLUSIONS

In summary, we for the first time successfully prepared unique BHCNs with an open mouth on their bottom by employing asymmetric B‐SiO_2_ NPs as a hard template, followed by the coating of PDA thin layer, subsequent carbonization of PDA and finally selective etching of SiO_2_. The asymmetric BHCNs could achieve self‐thermophoresis propulsion owing to the asymmetric thermal gradient generation under 808 nm NIR laser irradiation, which benefited from the combination of the asymmetric morphology and the excellent photothermal conversion ability of BHCNs. The motion of BHCNs‐X was directional with negative phototaxis, and the BHCNs‐15 had the fastest movement speed of ≈ 11.4 μm⋅s^−1^ in 20 s under 1.5 W⋅cm^−2^ of NIR laser irradiation. Furthermore, the BHCNs‐15 nanomotor exhibited higher removal efficiency of MB (53.4% vs. 25.4%) under NIR laser irradiation than that without NIR laser illumination. Overall, we developed a smart approach to fabricate the asymmetric carbon nanomotors equipped with bullet‐shaped, the hollow structure and an open mouth, which may open new horizons for its application in environment pollution, biomedical, biosensing, and other fields.

## CONFLICT OF INTEREST

The authors declare no conflict of interest.

## Supporting information

Figure. S1. A) The length and B) width size distribution of B‐SiO2 NPs.
Figure. S2. A–C) The TEM images of B‐SiO2@PDA‐X NPs prepared by different amounts of dopamine hydrochloride:
Figure. S3. A–C) The high resolution XPS spectra of C 1s, N 1s, and O1s of BHCNs‐5, respectively
Figure S4. A,E,I) The trajectories, B,F,J) De, C,G,K) speed and D,H,L) MSD of B‐SiO_2_ NPs, B‐SiO_2_@PDA‐5 and BHCNs‐5 nanomotors under different power destiny (0, 0.5, 1, 1.5 W cm^−2^) of NIR laser.
Figure S5. A) The schematic illustration of the comparison of the possible NIR light‐propelled mechanism of BHCNs‐5 and HMCNs nanomotors with different morphologies.
Figure S6. A,B) Scheme of the experimental setup for the observation of absorbance change of BHCNs‐15 aqueous suspension (500 μg/ml) with different incidence directions of UV–vis spectrophotometer under 808 nm NIR laser (1 W/cm^−2^) in 30 min and corresponding UV–vis absorption curves
Figure S7. A,D,G) The UV–vis absorbance spectra of different concentrations of MB (2, 10, 20 ug/ml) under different conditions
Figure S8. The UV–vis absorbance spectra of MB (5 μg/ml) under NIR laser irradiation with powder density of 1 W/cm^2^ under different irradiation times.

Video S1. Representative motion movies of B‐SiO_2_, B‐SiO_2_@PDA‐5 NPs, and BHCNs‐5 under the irradiation of NIR laser (1.5 W/cm^2^, 980 nm).

Video S2. Representative motion movies of BHCNs‐5, BHCNs‐10, and BHCNs‐15 under the irradiation of NIR laser (1.5 W/cm^2^, 980 nm).

Video S3. Representative motion movies of BHCNs‐15 under the irradiation of NIR laser (980 nm) with different power densities of 0, 0.5, 1, and 1.5 W/cm^2^.

## Data Availability

All data related to this work are present in the article and in Supporting Information. Any other data associated with this work are available from the corresponding authors upon request.
