# Supplementary material for: NIR light‐propelled bullet‐shaped carbon hollow nanomotors with controllable shell thickness for the enhanced dye removal
Source: Exploration (Beijing). 2022 Nov 18;2(6):20210162. doi: 10.1002/EXP.20210162 (PMC10191002; doi:10.1002/EXP.20210162)
Supplement: Supplementary file 1 — Figure. S1. A) The length and B) width size distribution of B‐SiO2 NPs. Figure. S2. A–C) The TEM images of B‐SiO2@PDA‐X NPs prepared by different amounts of dopamine hydrochloride: Figure. S3. A–C) The high resolution XPS spectra of C 1s, N 1s, and O1s of BHCNs‐5, respectively Figure S4. A,E,I) The trajectories, B,F,J) De, C,G,K) speed and D,H,L) MSD of B‐SiO2 NPs, B‐SiO2@PDA‐5 and BHCNs‐5 nanomotors under different power destiny (0, 0.5, 1, 1.5 W cm−2) of NIR laser. Figure S5. A) The schematic illustration of the comparison of the possible NIR light‐propelled mechanism of BHCNs‐5 and HMCNs nanomotors with different morphologies. Figure S6. A,B) Scheme of the experimental setup for the observation of absorbance change of BHCNs‐15 aqueous suspension (500 μg/ml) with different incidence directions of UV–vis spectrophotometer under 808 nm NIR laser (1 W/cm−2) in 30 min and corresponding UV–vis absorption curves Figure S7. A,D,G) The UV–vis absorbance spectra of different concentrations of MB (2, 10, 20 ug/ml) under different conditions Figure S8. The UV–vis absorbance spectra of MB (5 μg/ml) under NIR laser irradiation with powder density of 1 W/cm2 under different irradiation times. [file EXP2-2-20210162-s004.docx]

Supporting information

**NIR light-propelled bullet-shaped carbon hollow nanomotors with controllable shell thickness for the enhanced dye removal**

Jinyang Lv^a^, Yi Xing^a^, Xiaoyu Li^b^, Xin Du^a,^*

^a^ Beijing Key Laboratory for Bioengineering and Sensing Technology, School of Chemistry and Biological Engineering, University of Science and Technology Beijing, Beijing 100083, P. R. China.

***** E-mail: [duxin@ustb.edu.cn](mailto:duxin@ustb.edu.cn)

^b^ National Engineering Laboratory for Hydrometallurgical Cleaner Production Technology, Key Laboratory of Green Process and Engineering, Institute of Process Engineering, Chinese Academic of Sciences, Beijing 100190, China.

**Characterization**

For transmission electron microscopy (TEM) observation, sample was added the copper net and observed by HT7700 transmission electron microscope at an acceleration voltage of 60 kV. Scanning electron microscopy (SEM) images were carried out on a SU8010 scanning electron microscope. Composition and valence state of the samples were analyzed by X-ray photoelectron spectroscopy (XPS, ESCALAB 250Xi). UV-visible absorption spectra were carried out using a Shimadzu UV-1800 spectrophotometer. The analysis of hydrodynamic particle size was measured by dynamic light scattering (DLS) (Nano ZS, Malvern Instruments, Worcestershire, UK). Infrared thermal images were recorded using a thermal imager (TiS65, Fluke, USA).


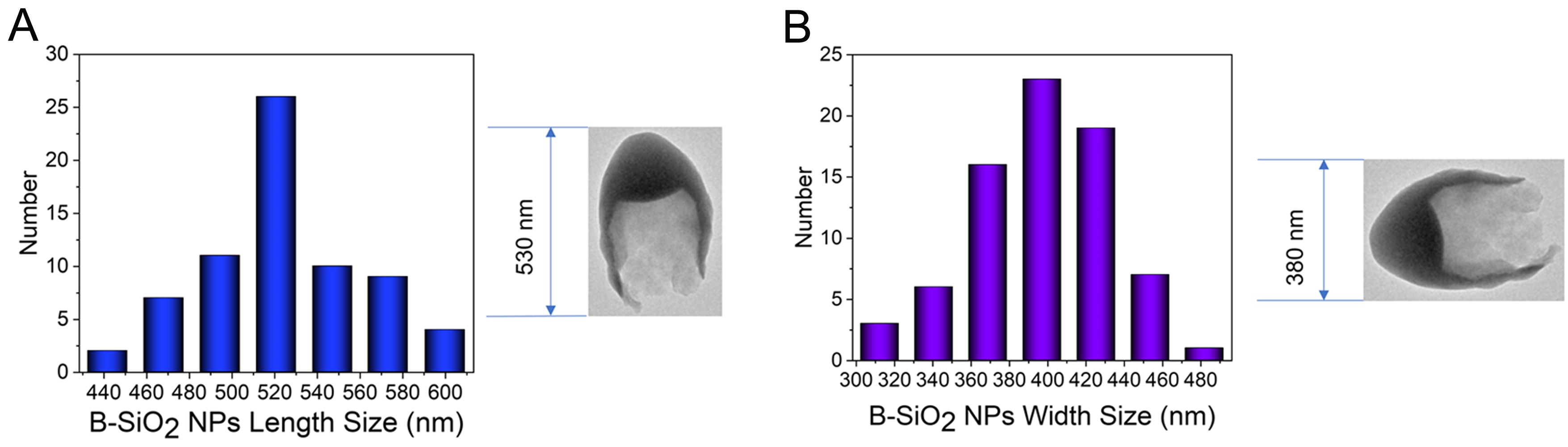


**Figure. S1.** A) The length and B) width size distribution of B-SiO_2_ NPs.


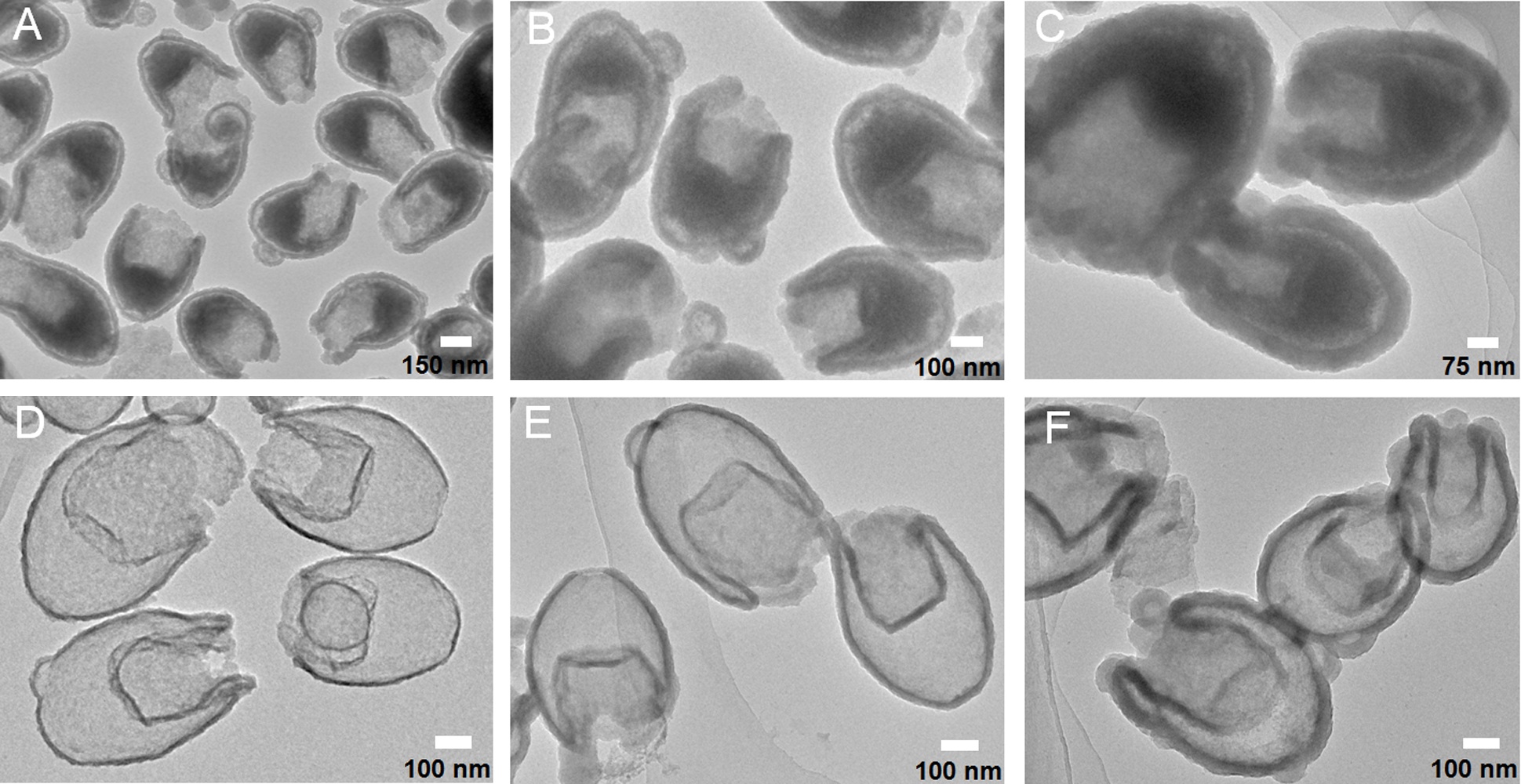


**Figure. S2.** A-C) The TEM images of B-SiO_2_@PDA-X NPs prepared by different amounts of dopamine hydrochloride: (A) 5, (B) 10 and (C) 15 mg. D-F) The TEM images of BHCNs-X.


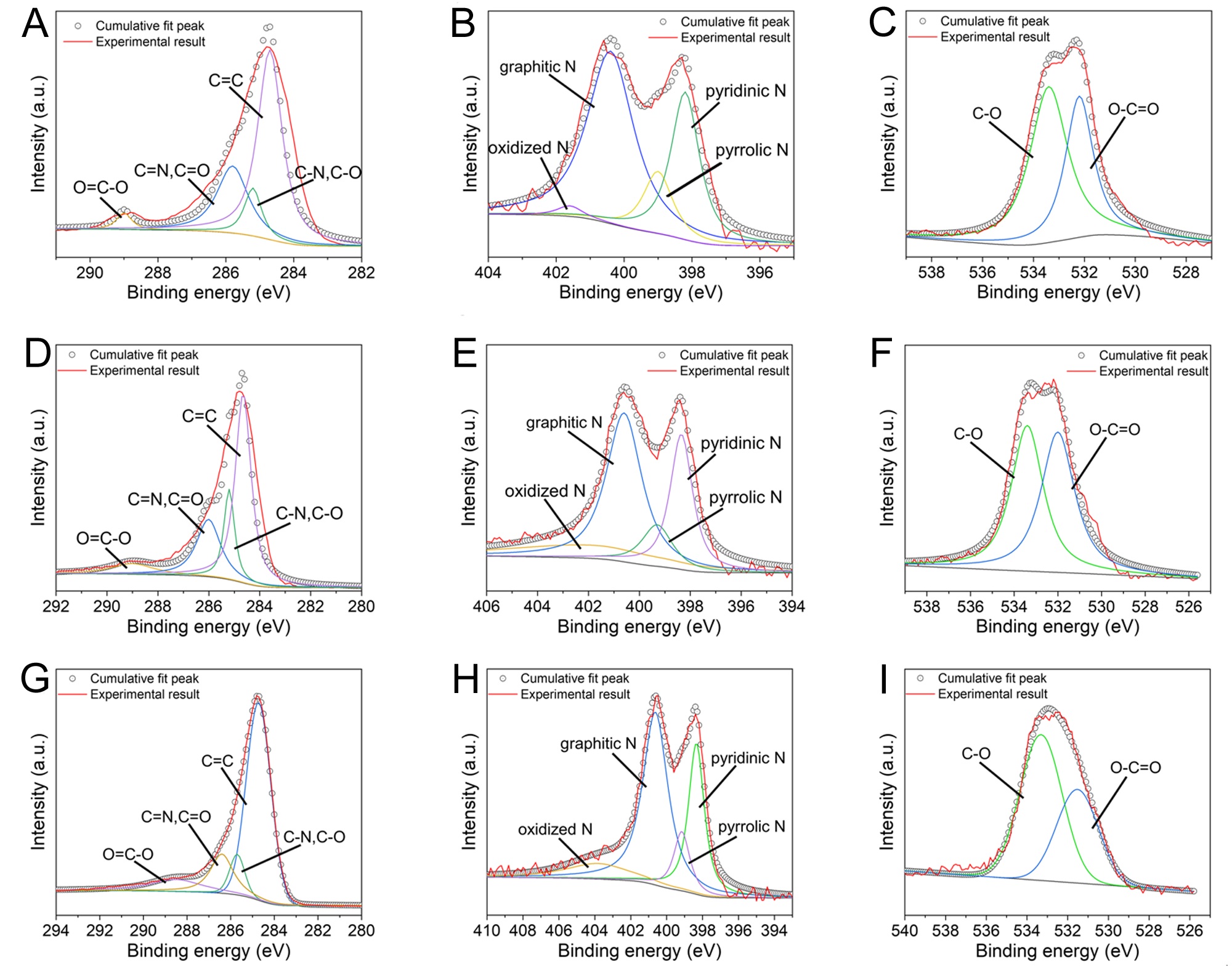


**Figure. S3.** A-C) The high-resolution XPS spectra of C 1s, N 1s and O1s of BHCNs-5, respectively. D-F) The high-resolution XPS spectra of C 1s, N 1s and O1s of BHCNs-10, respectively. G-I) The high-resolution XPS spectra of C 1s, N 1s and O1s of BHCNs-15, respectively.


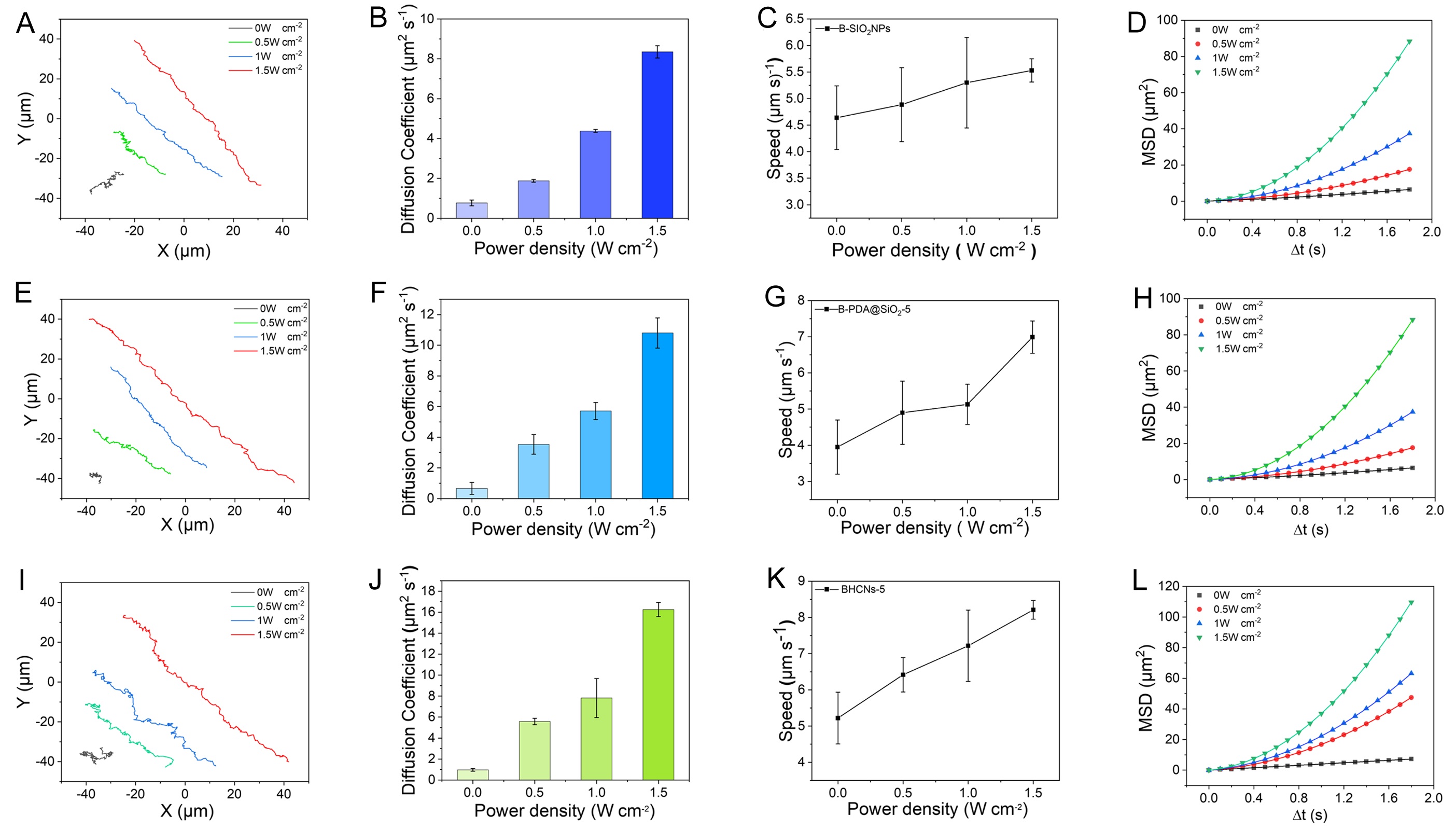


**Figure S4.** A, E, I) The trajectories, B, F, J) De, C, G, K) speed and D, H, L) MSD of B-SiO_2_ NPs, B-SiO_2_@PDA-5 and BHCNs-5 nanomotors under different power destiny (0, 0.5, 1, 1.5 W cm^-2^) of NIR laser.


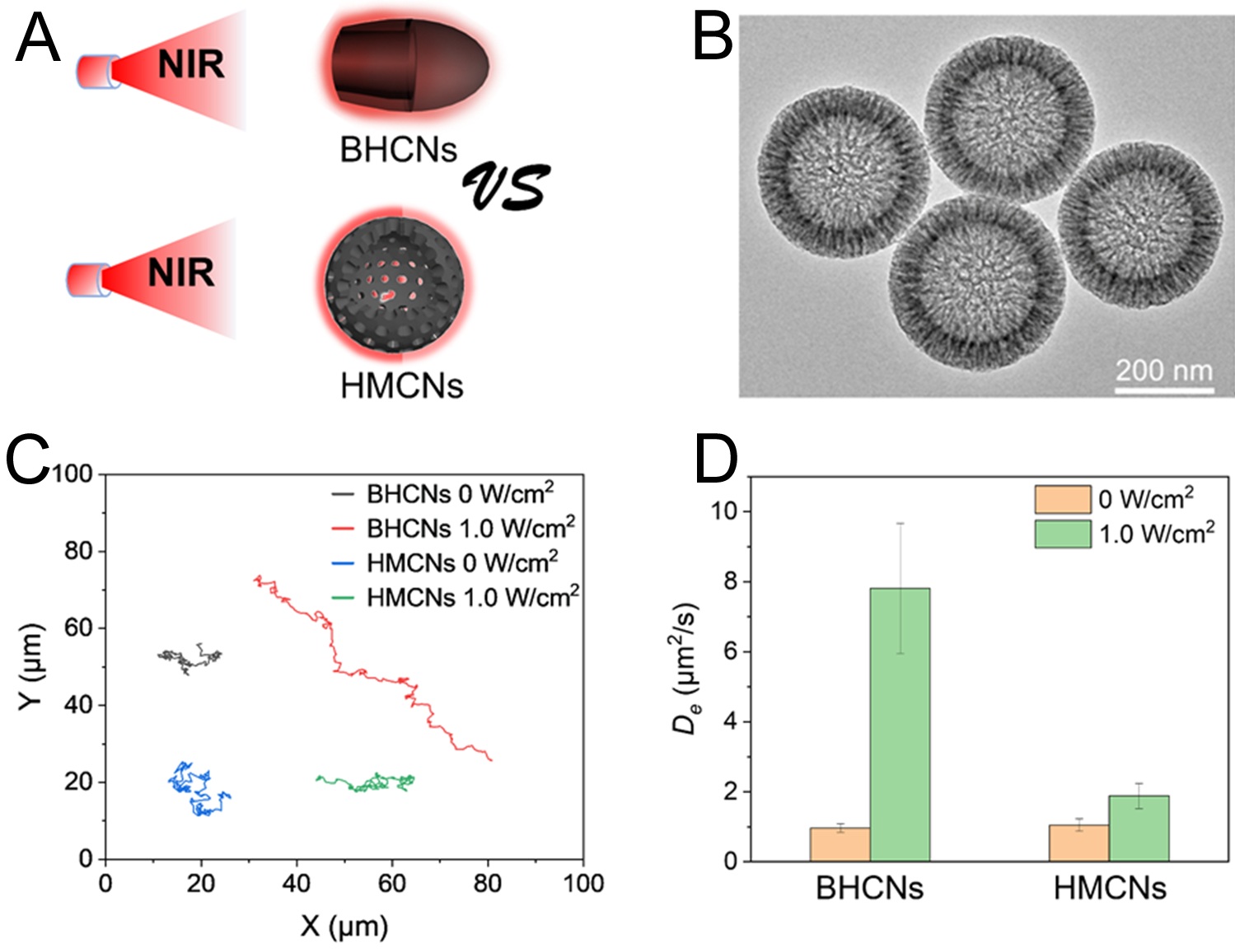


**Figure S5.** A) The schematic illustration of the comparison of the possible NIR light-propelled mechanism of BHCNs-5 and HMCNs nanomotors with different morphologies. B) The TEM image of HMCNs. C) Trajectories of HMCNs and BHCNs-5 nanomotors powered by 808nm NIR laser (0, 1.0 W/cm^2^). D) The *D_e_* of HMCNs and BHCNs-5 nanomotors under different power density (0, 1.0 W/cm^2^) of NIR laser.


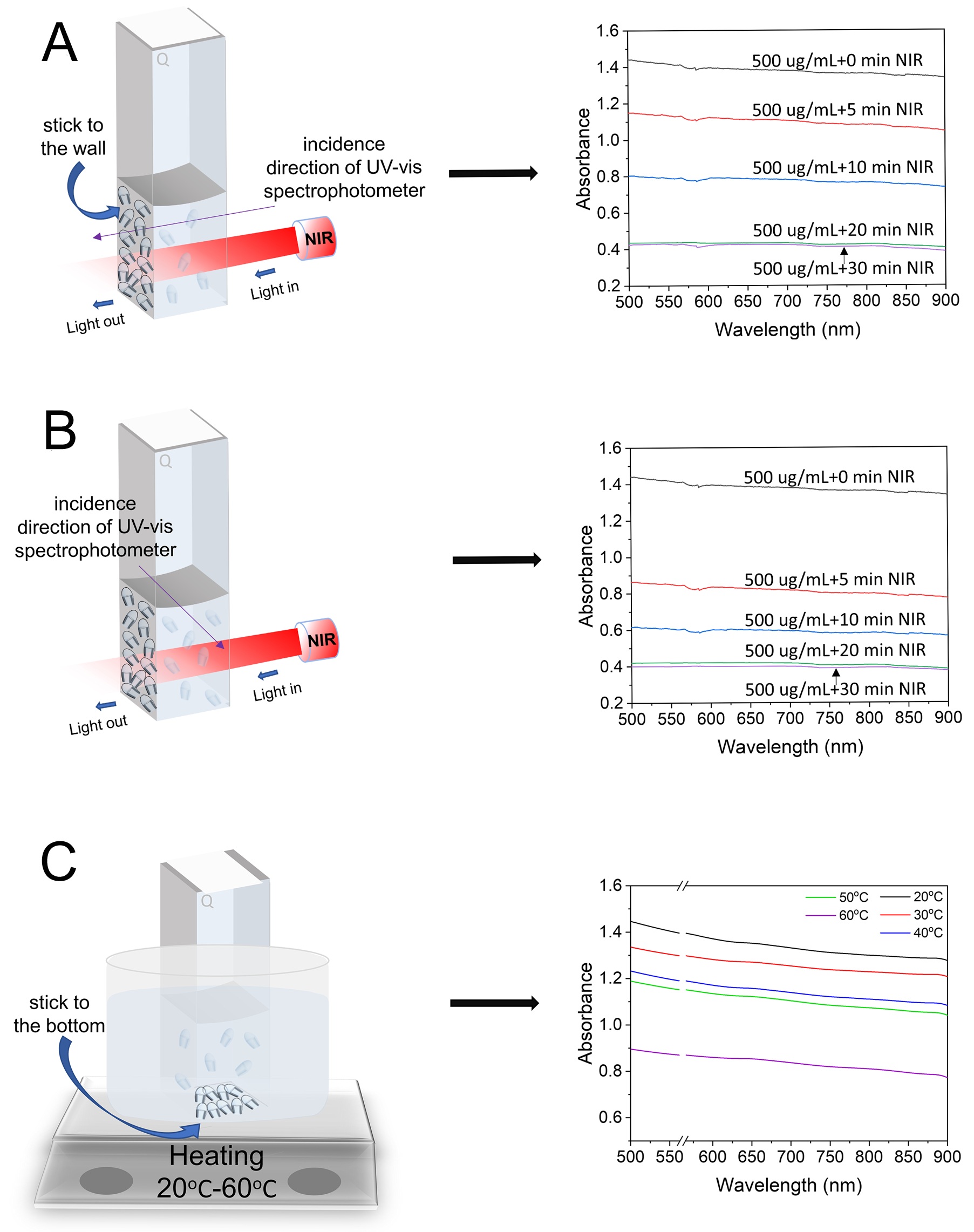


**Figure S6.** A, B) Scheme of the experimental setup for the observation of absorbance change of BHCNs-15 aqueous suspension (500 μg/mL) with different incidence directions of UV-vis spectrophotometer under 808nm NIR laser (1 W/cm^-2^) in 30 min and corresponding UV-vis absorption curves. C) Scheme of the experimental setup for the observation of absorbance change of BHCNs-15 aqueous suspension (500 μg/mL) under different temperature and corresponding UV-vis absorption curves.


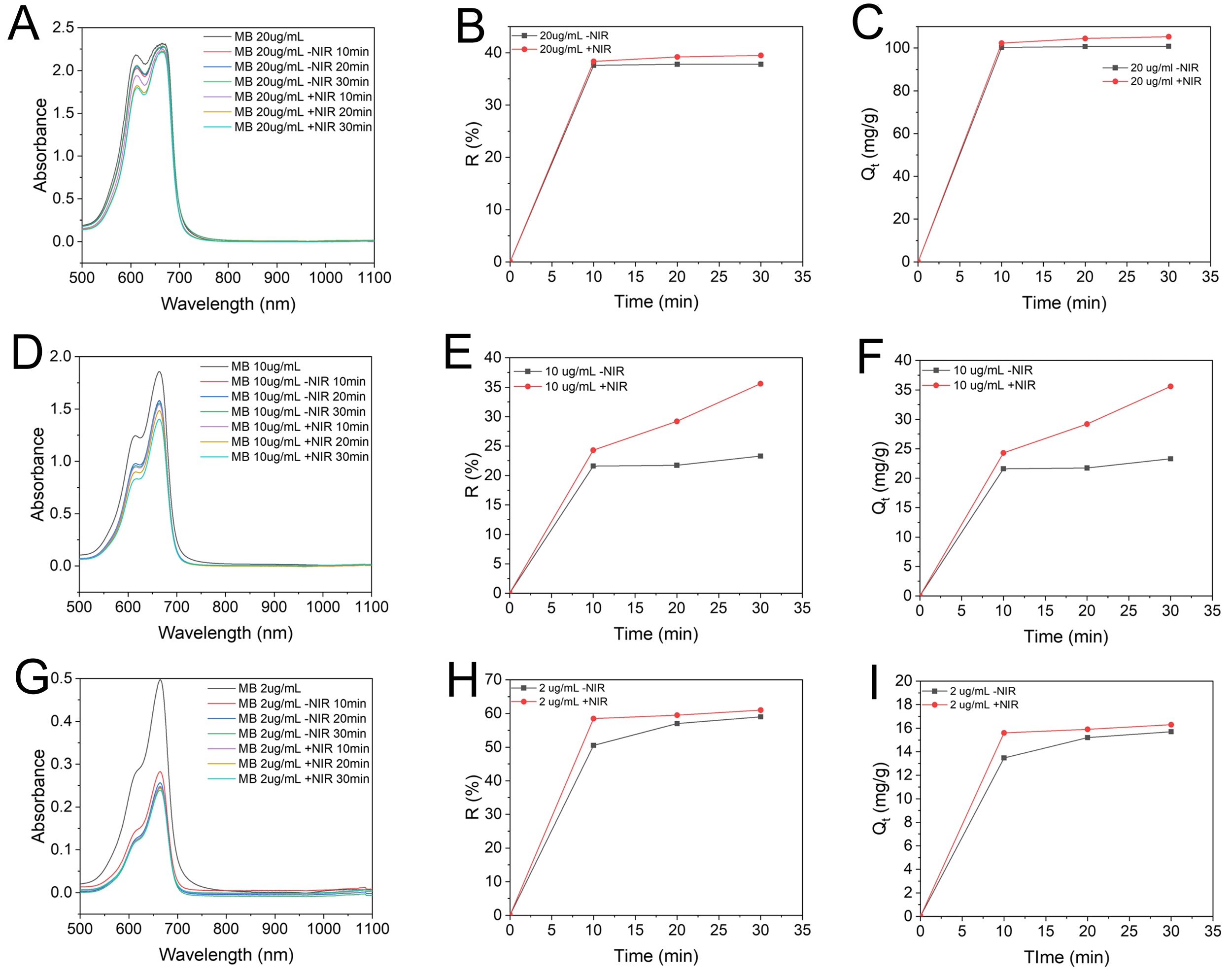


**Figure S7.** A, D, G) The UV-vis absorbance spectra of different concentrations of MB (2, 10, 20 ug/mL) under different conditions: static BHCNs-15 micromotors (-NIR) and dynamic BHCNs-15 micromotors (+NIR). B, E, H) Time-dependent changes of removal efficiency for different concentrations of MB (2, 10, 20 ug/mL) under different conditions: static BHCNs-15 micromotors (-NIR) and dynamic BHCNs-15 micromotors (+NIR). C, F, I) Time-dependent changes of adsorption capacity for different concentrations of MB (2, 10, 20 ug/mL) under different conditions: static BHCNs-15 micromotors (- NIR) and dynamic BHCNs-15 micromotors (+NIR).


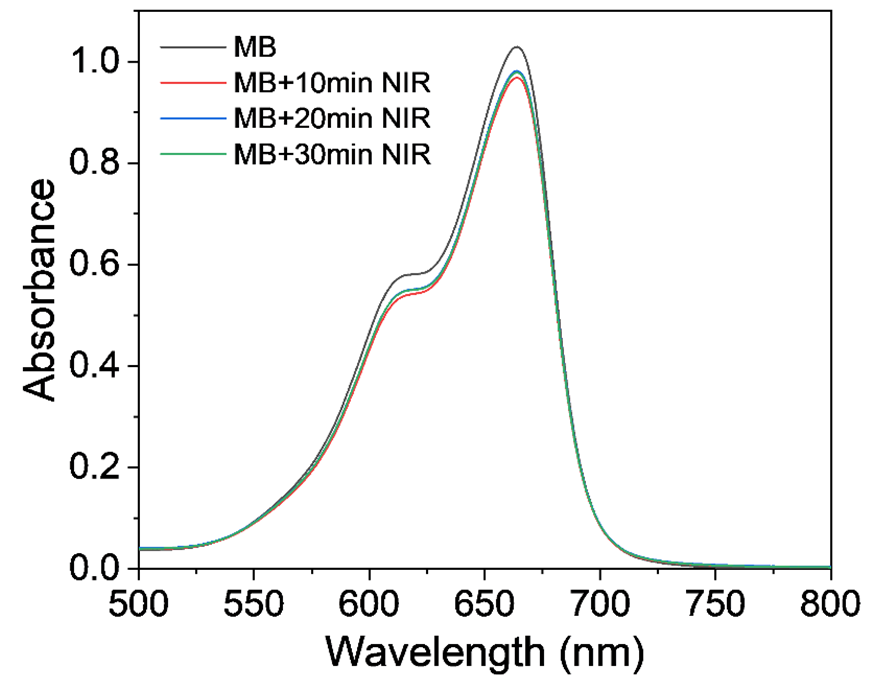


**Figure S8.** The UV-vis absorbance spectra of MB (5 μg/mL) under NIR laser irradiation with powder density of 1 W/cm^2^ under different irradiation time.

Video S1. Representative motion movies of B-SiO_2_, B-SiO_2_@PDA-5 NPs and BHCNs-5 under the irradiation of NIR laser (1.5 W/cm^2^, 980 nm).

Video S2. Representative motion movies of BHCNs-5, BHCNs-10 and BHCNs-15 under the irradiation of NIR laser (1.5 W/cm^2^, 980 nm).

Video S3. Representative motion movies of BHCNs-15 under the irradiation of NIR laser (980 nm) with different power density of 0, 0.5, 1 and 1.5 W/cm^2^.
